# The Effect of Team Job Crafting on Professional Quality of Life Among Nurses: A Latent Profile Analysis

**DOI:** 10.1155/jonm/2320459

**Published:** 2025-07-15

**Authors:** Xu-Hua Zhou, Di-Fei Duan, Lin Chen, Ying-Jun Zhang, Shu Gong, Qian Chen

**Affiliations:** ^1^Hemodialysis Center, Department of Nephrology, West China Hospital, Sichuan University/West China School of Nursing, Sichuan University, Chengdu 610213, Sichuan, China; ^2^Department of Nephrology, Kidney Research Institute, West China Hospital, Sichuan University/West China School of Nursing, Sichuan University, Chengdu 610213, Sichuan, China; ^3^Nursing Department, West China Hospital, Sichuan University/West China School of Nursing, Sichuan University, Chengdu 610213, Sichuan, China; ^4^Center of Gerontology and Geriatrics, West China Hospital, Sichuan University/West China School of Nursing, Sichuan University, Chengdu 610213, Sichuan, China

## Abstract

**Aims:** This study aimed to classify the latent profiles of team job crafting of nurses and to examine their effect on professional quality of life (ProQOL).

**Background:** Team job crafting, as an emerging concept, could promote team productivity and performance by enhancing collaboration among team members and might affect nurses' ProQOL. However, whether different population characteristics of team job crafting for nurses pattern latent profiles and how these subgroups correlate with nurses' ProQOL remains to be determined.

**Methods:** This cross-sectional study was conducted at a general tertiary hospital in Sichuan Province, China. Two thousand two hundred fifty-three nurses completed an online investigation encompassing the Team Job Crafting Scale for Nurses and the Professional Quality of Life Scale. Latent profile analysis and hierarchical regression analysis were employed to validate our research hypotheses.

**Results:** The team job crafting of nurses was classified into two subgroups: “poor team job crafting group” (*n* = 973, 43.2%) and “excellent team job crafting group” (*n* = 1280, 56.8%). The different profiles of team job crafting exhibited significant effects on three subvariables of nurses' ProQOL: compassion satisfaction (adjusted *R*^2^ = 0.208, *p* < 0.001), burnout (adjusted *R*^2^ = 0.192, *p* < 0.001), and secondary traumatic stress (adjusted *R*^2^ = 0.043, *p* < 0.001).

**Conclusions:** Team job crafting for nurses was characterized by a strong population heterogeneity. The majority of nurses perform excellent in the team job crafting. The team job crafting was significantly associated with the ProQOL among nurses. Nursing managers should draw up targeted continuing educational training programs and organizational policies to promote the positive participation of nurses in team job crafting, thereby improving their ProQOL.

## 1. Introduction

Nurses hold a vital position in the global healthcare system, not only taking care of patients on a routine basis but also carrying out multiple roles such as emotional support, health education, and coordination of medical care [1–3]. However, with an aging global population and an increasingly complex disease spectrum, the demand for healthcare keeps increasing, directly contributing to the increased workload of nurses [4]. Intensive workloads make the nursing community vulnerable to problems such as compassion fatigue (CS), burnout (BO), and secondary traumatic stress (STS) [5–7]. These concerns not only directly affect nurses' professional quality of life (ProQOL) but also indirectly hurt the quality of healthcare services and patient health outcomes [8–10]. Therefore, how to bring about an enhanced working environment for nurses and improve their ProQOL has become an urgent and important agenda in the field of nursing management.

The ProQOL is a widely applied instrument for evaluating the psychological wellbeing of professionals in the workplace, specifically measuring the CS, BO, and STS [11, 12]. These positive and negative factors interfere with the workforce's psychological balance and wellbeing, especially among the community of nurses [13]. CS is a positive component of assessing the ProQOL among nurses and positively affects their job wellbeing and satisfaction [14, 15]. However, a previous meta-analysis that included 28,509 nurses indicated that the CS of nurses worldwide was not optimistic, especially in Asia [16]. In addition, as the negative aspects of ProQOL, the global prevalence of BO and STS among nurses remains consistently high [17]. According to statistics, the prevalence of BO among nurses in intensive care units was 50% [18]. Furthermore, approximately 65% of emergency nurses worldwide suffer from STS [19]. More critically, both BO and STS were associated negatively with nurses' occupational mental health and patient health outcomes in the organizational environment and clinical care [20, 21]. These data and results highlight the urgency of improving nurses' ProQOL. However, previous studies have demonstrated that there are a variety of factors affecting nurses' ProQOL, typically including individual characteristics, organizational policies and the external environment [12, 17], of which organizational and external environmental factors are of critical concern to nursing managers and could be improved by optimizing organizational policies. This highlights the significance of further investigating the impact of novel variables in changeable organizational domains on the ProQOL.

As a crucial variable in the organizational field, team job crafting originated from job crafting proposed by Wrzesniewski and Dutton in 2001 [22] and is an emerging concept with significant theory and practice implications. It was defined as strengthening team productivity, cohesion, and the psychological wellbeing of its members by rationalizing how team members collaborate and adapting their structure and workflows [23]. More critically, team job crafting represents an actionable and modifiable factor within the immediate work environment. By concentrating on this aspect, nursing managers and policymakers with practical strategies directly improve nurses' wellbeing at the team level. With the evolution of modern medicine, multidisciplinary teamwork in healthcare has gradually become the dominant model in the field of hospital care [24]. In this context, team job crafting for nurses is more essential than job crafting at the individual level. Since confronted with this change in the organizational environment, members of the care team have to establish a common goal orientation and adopt more positive interactions with the team to achieve greater efficiency and quality of care [25]. Nevertheless, most studies focus on individual job crafting, overlooking team dynamics in healthcare settings. Moreover, team job crafting was demonstrated to be positively correlated with work engagement among nurses [26]. However, few studies evaluated the team job crafting of nurses and its impact on their ProQOL, and the only earlier study distinguished the extent of team job crafting by scale scores, ignoring the heterogeneity of the population, which may have led to imprecise estimates. Thus, an attempt was undertaken to introduce a human-centered approach for latent profile analysis (LPA) [27] to achieve an accurate categorization of the team job crafting in nurses.

Given its usefulness in supporting the comprehension of the impact of various job features on professional wellbeing, the Job Demands-Resources (JD-R) model has become one of the favored theoretical models of nursing management [28]. The JD-R model posits that occupational health outcomes are shaped by the interplay between job demands and job resources [29]. The JD-R model encompasses both job demands and job resources, which interact with each other to achieve a dynamic balance between an individual's physical and psychological wellbeing [29, 30]. In the context of nursing, team job crafting (proactive adjustments to job demands and resources) reflects the JD-R model's premise, as it helps nurses optimize their work environment, reducing BO and promoting compassion satisfaction [31]. Specifically, according to the JD-R model, Tims described team job crafting as behavioral changes made by team members to counterbalance job demands and resources according to their preferences of requirements to promote occupational health [31]. Therefore, we consider that team job crafting may have a significant effect on nurses' ProQOL (a subset of occupational health). Drawing on the above literature, this study aimed to categorize the latent profile of team job crafting of nurses and to examine its impact on the ProQOL.

## 2. Methods

### 2.1. Study Design, Setting, and Participants

This study utilized a cross-sectional design and recruited clinical nurses using a convenience sampling method. Participants were selected from a general tertiary hospital in Sichuan Province, China, which employs approximately 5000 nursing staff, between January and March 2024. The inclusion criteria included (1) registered nurses with nationally certified professional license; (2) employment duration ≥ 1 year (to ensure participants had sufficient exposure to team workflows and stabilized ProQOL assessments); and (3) volunteering to participate in the study. Exclusion criteria comprised nurses who were off duty during the survey period due to reasons such as pregnancy, illness, or personal matters.

### 2.2. Measurements

#### 2.2.1. Demographic Information Form

The structured questionnaire comprises eight demographic variables: gender, age, marital status, number of children, education level, working years, working area, and rotational night shift.

#### 2.2.2. Team Job Crafting Scale for Nurses (TJCS-N)

The TJCS-N, complied by Iida [23] and translated into Chinese by Fang [32], is a 13-item scale designed to measure nurses' perceived team job crafting. Each item is scored from 1 to 5 (1 = not at all and 5 = extremely). The initial version of TJCS-N consists of three dimensions: “task crafting,” “cognitive crafting,” and “relational crafting.” The Chinese version of TJCS-N incorporated the two subdimensions of “cognitive crafting” and “relational crafting” into one subdimension and renamed it “cognitive and relational crafting.” The Cronbach's α for the original scale and the Chinese version were 0.91 and 0.95, respectively [23, 32]. In this study, the TJCS-N demonstrated adequate reliability indices (Cronbach's α = 0.886).

#### 2.2.3. Professional Quality of Life Scale

The scale developed by Stamm [33] and revised by Zheng [34] is a 30-item, 5-point Likert scale, and it was utilized to assess nurses' ProQOL in this study. The scale covers three dimensions: “compassion satisfaction,” “BO,” and “STS.” A previous study reported a Cronbach's α of 0.91 for the Chinese version of the scale and a Cronbach's α ranging from 0.73 to 0.87 for its three subscales [34]. This study revealed that the scale remained at an acceptable level of reliability with a total Cronbach's α of 0.72.

### 2.3. Data Collection

Thirty-five trained nurse leaders were allocated as investigators and coordinators, performing four key roles: (1) disseminating survey links through authorized WeChat work groups; (2) conducting participant briefings on study objectives and procedures; (3) verifying preliminary eligibility; and (4) providing technical support during the 2-weeks data collection period. The link to the questionnaire was posted through the Questionnaire Star platform, and the investigators distributed the hyperlink to the working WeChat group and explained in detail the purpose and significance of the study and the cautions for completion. At the beginning of the online questionnaire, we clearly presented all inclusion criteria. Participants were explicitly informed that they must meet all criteria to proceed with the survey. They were asked to confirm their eligibility by clicking an “I agree” button before accessing the main content of the questionnaire. For quality assurance, each IP address was restricted to submitting only one questionnaire, and all questions have been set as mandatory. In addition, a pretest including 20 nurses was implemented to examine the time required to complete the questionnaire, and a total answer time of 15 min was ultimately determined. The survey data were checked and purified by two researchers, and the questionnaires with excessive or short response times and regular answers were excluded. To be specific, one nursing management specialist was responsible for clinical soundness review; one master's degree in nursing was responsible for technical quality control. In addition, any questionnaires with inconsistent the inclusion criteria were excluded from the final analysis. During recruitment, 312 nurses were excluded due to having < 1 year of work experience. A total of 2720 questionnaires were distributed in this study and 2253 valid questionnaires were recovered, with a valid response rate of 82.8%. Specifically, a total of 467 questionnaires were excluded during subsequent data cleaning. The exclusion criteria and corresponding proportions were as follows: insufficient work experience (20 cases, 4.3%), abnormally long or short response times (213 cases, 45.6%), and patterned responses (234 cases, 50.1%).

### 2.4. Statistical Analysis

The LPA of team job crafting was conducted using Mplus 8.3, based on the scores of each entry of the Chinese Version of the Team Job Crafting Scale for Nurses, and the optimal number of profile classifications was selected by gradually increasing the profiles according to the fit indices test. The test indicators included the Akaike information criterion (AIC), Bayesian information criterion (BIC), and adjusted BIC (aBIC); the smaller the fit index, the better the model fitting effect. The entropy value (Entropy) was employed to determine the classification accuracy of the model when it is ≥ 0.8 and when nearing to 1, it suggested that the latent profiles are more credible [35]. When the *p* value corresponding to the Lo–Mendell–Rubin test (LMRT) and bootstrap likelihood ratio test achieved a significant level (*p* < 0.05), it indicated that the model with *k* profiles outperformed the model with *k* − 1 profiles [36]. Data were analyzed using SPSS 27.0 software. Continuous material was expressed as the mean and standard deviation, while categorical variables were described using frequencies and percentages. The independent samples *t*-test and one-way analysis of variance (ANOVA) were implemented to detect differences between groups. Hierarchical linear regression was utilized to determine the effect of team job crafting on the professional quality of life. *p* < 0.05 was considered statistically significant (two-sided).

## 3. Results

### 3.1. Characteristics of Participants

A total of 2253 questionnaire data were included in the statistical analysis (Table 1). The majority of the surveyed population was female (93.4%), with only 6.6% male. The age of the participants ranged from 20 to 59, with a mean age of 34.01 ± 7.07 years. Most nurses were married (69.7%) and only 2.5% were divorced or widowed. The overwhelming number of participants was parenting one or even more children. In addition, the majority had an undergraduate diploma (86.7%), 8.5% had a graduate degree, and 5.1% held a junior college degree. The working years of the nurses varied from 1 to 40, with a mean of 12.01 ± 7.58 years. In terms of the working area, the participants came from internal medicine (32.8%), surgery (23.3%), intensive care unit (18.3%), and other wards (25.6%). Furthermore, 62.6% of these nurses were still engaged in rotational night shifts.

### 3.2. Latent Profile of Team Job Crafting Among Nurses

**Figure 1:**
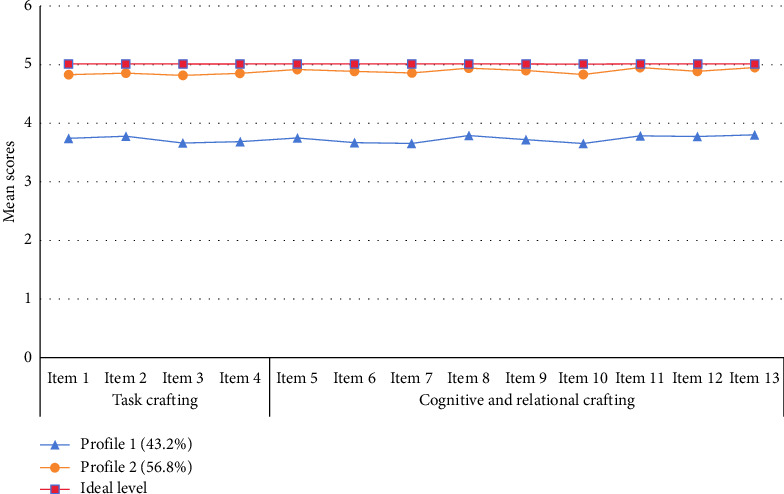
Latent profile analysis result of the team job crafting.

Based on the results of the evaluation of each item of team job crafting among nurses, we fitted three latent profile models (Table 2). AIC, BIC, and aBIC decreased gradually as the number of categories increased. When the number of model categories was 3, both LMRT and BLRT tests did not achieve a significant level (*p* > 0.05), so Profile 2 was determined to be the best-fit model. The 13-item scores corresponding to the 2 latent profiles of team job crafting in nurses are presented in Figure 1. According to the questionnaire scoring criteria, if the nurse's team job crafting is considered perfect, the ideal score across each item should be five points. The mean scores in each item for Profile 1 were notably below the ideal and significantly lower than those of Profile 2. Thus, it was designated as the “poor team job crafting group.” In Profile 2, scores across all items approached the ideal. Consequently, this group was labeled the “excellent team job crafting group,”. “poor team job crafting group,” and “excellent team job crafting group,” which comprised 43.2% (*n* = 973) and 56.8% (*n* = 1280) of the overall participants, respectively.

### 3.3. Comparison of ProQOL Among Nurses Across the Two Profiles

There was a statistically significant difference between the scores of the two latent profile patients on the three dimensions of CS, BO, and STS (all *p* < 0.001). Specifically, Profile 1 had significantly lower CS scores but higher BO and STS as compared with Profile 2. The results of the comparison of CS, BO, and STS scores for the two profiles are detailed in Table 3.

### 3.4. Comparison of ProQOL Among Nurses in Demographic Characteristics

Univariate analysis was employed to compare differences in nurses' professional quality of life between demographic characteristics (Table 4). The results indicated statistically significant differences in CS across age, marital status, number of children, education level, working years, working area, and rotational night shift. These variables were subsequently included as covariables in the regression analysis (Table 5) to isolate the independent effect of profile membership of team job crafting. In addition, BO was observed to be significantly different for all demographic variables of interest. Furthermore, STS was identified to differ in terms of gender, working area, and rotational night shift.

### 3.5. The Effect of Latent Profiles of Team Job Crafting on ProQOL

Demographic variables determined by statistical significance were set as dummy variables and the first item was used as a reference. After considering the control variables, we validated the study hypotheses using hierarchical regression analyses (Table 5). The results demonstrated that team job crafting with different latent profiles exerted a significant positive effect on nurses' CS (Profile 1 vs. Profile 2, *β* = 0.466, *p* < 0.001). Moreover, compared with Profile 1, Profile 2 exhibited a significantly negative impact on BO (*β* = −0.449, *p* < 0.001) and STS (*β* = −0.211, *p* < 0.001).

## 4. Discussion

This study identified different latent profiles of team job crafting among nurses and examined their effect on indicators of ProQOL including CS, BO, and STS. The results indicated that team job crafting of nurses could be classified into two latent categories and had a significant positive effect on CS and a negative predictive role for BO and STS. To our knowledge, the present study constitutes the first attempt to identify the potential classes of team job crafting among nurses using latent profile analysis and determine the effect of these profiles on the ProQOL. This study provides new insights into the appropriate understanding of nurses' corps on job reshaping and robustly confirms the significant impact of team job crafting on the ProQOL, which may contribute to the developing of desirable organizational management strategies for nursing managers to improve the ProQOL of nurses.

Our findings revealed that team job crafting among nurses can be categorized into “poor team job crafting group” (43.2%) and “excellent team job crafting” group” (56.8%) based on the results of model fitting. These results suggested that the majority of nurses performed outstandingly in team job crafting. Given the fact that nurses, as a highly specialized workforce, are responsible for the health of their patients, nursing teams generally exhibit a clear delineation of individual roles and responsibilities, and this structure allows team members to adapt more positively to the challenges and pressures of the workplace [37, 38]. Moreover, within the clinical work setting, nurses are required to collaborate intimately with other multidisciplinary team members to facilitate patient health outcomes, which equips them with a good sense of teamwork and the ability to modify themselves and the team's working model in a timely manner to accommodate changes in the workplace situation [39]. Finally, the overall working years of the participants in this study had been longer, and their substantial experience in clinical work enabled them to be comfortable in improving nursing workflow and coordinating interpersonal relationships. As a result, hospital administrators should provide training programs for young nurses to facilitate team job crafting, enhance their team management and coordination skills, and thereby improve work efficiency.

Compassionate satisfaction (CS) is defined as the feeling of contentment, wellbeing, and achievement experienced by an individual in the process of offering assistance or care to patients and constitutes a positive element among the crucial components of the ProQOL [14]. Our study observed a significant positive association between team job crafting and CS among nurses. According to the Conservation of Resources Theory (COR), individuals maintain their inner psychological energy by acquiring, conserving, and investing in the resources [40]. Established research suggests that prolonged exposure to high-stress teamwork environments significantly depletes nurses' psychological resources-including structural resources such as role optimization to reduce ambiguity, social resources like coworker support for emotional replenishment, and cognitive resources such as shared mental models to enhance work meaning which, if not effectively replenished, may diminish their CS [41]. Preliminary research has shown that team job crafting can promote communication and collaboration by optimizing the assignment of roles and enhancing support and trust among team members [19]. Nurses in a supportive group setting are more effective at splitting workloads, reducing fatigue and emotional burdens, and thus investing more energy in compassionate patient care [42]. More specifically, team job crafting may optimize the allocation of team resources through structural role optimization (task, cognitive, and relational crafting) and reduce the cognitive load caused by role ambiguity, thus preserving emotional energy for the protection of nurses' CS. Therefore, hospital administrators could make full use of their leadership, refine the distribution of teamwork, and reinforce emotional connection and communication so that nurses might enjoy more professional achievement and affective fulfillment in the process of providing nursing care to patients.

BO is characterized by individuals suffering from prolonged stress, fatigue, and emotional exhaustion at work, resulting in decreased motivation and negative attitudes toward work [18]. It usually manifests itself in environments that are overloaded with work, chronically stressed, and lacking in adequate support [43]. BO was selected in our study as a negative factor in the assessment of ProQOL in nurses. Our findings showed that team job crafting is significantly and negatively associated with nurses' BO. Self-Determination Theory argues that the gratification of the three essential psychological needs of autonomy, competence, and relationships is critical to an individual's intrinsic motivation and psychological wellbeing [44]. The teamwork reinvention tended to empower nurses to reframe their roles in teamwork, opting for tasks that were more challenging and personally rewarding [26]. This change gives nurses a sense of being more meaningful on the team, which leads to more motivation and less BO [31]. Furthermore, another possible explanation is that within a well-organized teamwork environment, nurses could proactively adjust their affective engagement and pace of work to find room for recovery in a high-intensity work environment [45]. This adaptation enables them to find a better balance between work and rest, thus reducing the risk of emotional exhaustion [46]. When mental necessity has been fulfilled, nurses' intrinsic motivation and sense of wellbeing at work are strengthened, thus effectively alleviating BO problems such as physical and mental exhaustion and emotional depletion arising from prolonged work pressure [44]. Thus, nurse leaders would be expected to make relentless efforts to mitigate BO by creating a supportive and open work atmosphere, increasing staff autonomy and engagement, and optimizing task allocation.

Stress Theory holds that individuals who are faced with a marker of stress and are unable to cope with it with their own resources may experience a strain response [19]. STS is identified as a psychological stress response that occurs in the carer themselves as a result of continued exposure to the trauma narrative or in the face of the traumatized person's emotional distress [19]. In our study, STS was found to have a negative correlation with team job crafting, a relationship that can be elucidated through the lens of Stress Theory. From the perspective of stressors, team job task crafting allows nurses to select tasks that match their expertise and interests, effectively reducing exposure to trauma and laden situations [47]. This proactive task selection mechanism decreases the intensity and frequency of stressors, thereby mitigating the risk of STS, as per the Stress Theory's emphasis on the role of stressor management in preventing strain [48]. In addition, regarding coping resources, the process of team relationship remodeling enriches nurses' social support systems [49]. By sharing experiences and emotional resonance within the team, nurses can collectively develop coping strategies for traumatic work components [50]. This social support acts as a crucial coping resource, enhancing nurses' ability to deal with stress, which is consistent with Stress Theory's assertion that sufficient coping resources buffer the impact of stressors. In light of the above, nursing managers should train nurses on team job crafting including emotional regulation, interpersonal communication, and time management to facilitate their comprehension of how to adjust their roles, tasks, and work styles to better cope with stress and trauma exposure.

### 4.1. Limitations

Some limitations should be considered. First, the cross-sectional design of this study may limit the ability to establish reliable causal relationships between team job crafting and ProQOL. Second, as all samples were obtained from a single teaching hospital, the generalizability of the findings is limited. Third, the results of the assessments of team job crafting and ProQOL were derived from self-reports of nurses and the questionnaires were administered by the nurse managers, which could be subject to potential reporting bias. Finally, there are also numerous factors that influence nurses' ProQOL, and although team job grafting has been shown to be potentially strongly associated with ProQOL, further exploration of the mechanisms by which the different variables in the onset of the relationship interact are required.

### 4.2. Implications

In the context of the increasing complexity of nursing care, nurses work under tremendous pressure and their ProQOL is being challenged. The findings of this study carry critical implications for improving nurses' ProQOL. First, this study accurately identifies the different latent profiles of team job crafting among nurses, which holds a critical value for nursing managers in creating interventions focused on the characteristics of the population. Moreover, the significant effect of team job crafting on the ProQOL among nurses highlights the importance of incorporating appropriate training components into continuing education programs of nurses. Nursing managers could construct comprehensive team job crafting training programs and organizational policies based on our findings that not only focus on teamwork task coordination but also aim to promote harmonious interpersonal relationships in the team, which may be effective in improving the ProQOL of nurses.

To achieve effective team job crafting among nurses, organizational, human resources, wellbeing, and environmental factors must be systematically coordinated. First, the hierarchical structure should be optimized to improve decision-making efficiency, and an open and collaborative organizational culture should be actively cultivated alongside the clarification of strategic goals and plans for team job crafting. Second, the human resources department should focus on recruiting individuals who can collaborate and innovate as a team and on setting up a performance-oriented assessment and incentive system to motivate nurses. Third, workloads should be reduced through rational manpower allocation, the implementation of a pressure management system, and the promotion of healthy lifestyles. Finally, upgrading information technology will create a platform for intelligent collaboration and multifactor linkage, reshaping teamwork among nurses and providing comprehensive support.

## 5. Conclusions

In conclusion, team job crafting of nurses could be classified into two profiles: “poor team job crafting group” and “excellent team job crafting group.” In addition, the different categories of team job crafting exerted a significant effect on the nurses' ProQOL. Specifically, the team job crafting was a significant positive predictor of CS but had statistically significant negative impacts on BO and STS. These results may furnish nursing managers with valuable information for identifying the different categories of team job crafting among nurses and for tailoring interventions to improve their ProQOL.

## Figures and Tables

**Table 1:** Characteristics of participants (*N* = 2253).

Variables	*N*	%
*Gender*		
Male	149	6.6
Female	2104	93.4

*Age (years): 34.01 ± 7.07 (20–59)*		
18–30	758	33.6
31–45	1362	60.5
46–60	133	5.9

*Marital status*		
Unmarried	626	27.8
Married	1570	69.7
Divorced/widowed	57	2.5

*Number of children*		
0	937	36.9
1	1034	45.9
2	381	16.9
3	7	0.3

*Education level*		
Junior college	114	5.1
Undergraduate	1954	86.7
Graduate	185	8.2

*Working years: 12.01 ± 7.58 (1–40)*		
1–10	1017	45.1
11–20	982	43.6
21–30	189	8.4
31–40	65	2.9

*Working area*		
Internal medicine	739	32.8
Surgery	524	23.3
Intensive care units	413	18.3
Other wards	577	25.6

*Rotational night shift*		
Yes	1411	62.6
No	842	37.4

**Table 2:** Latent profile analysis of the team job crafting among nurses with fit indices.

Model	AIC	BIC	aBIC	Entropy	LMR	BLRT	Categorical probability
1	70384.092	70532.789	70450.183	—	—	—	—
**2**	**52600.368**	**52829.168**	**52702.082**	**0.975**	**0.0189**	**0.0195**	**0.432/0.568**
3	39528.148	39837.029	39665.46	0.988	0.0992	0.1007	0.073/0.393/0.534

*Note:* Entropy, entropy test; LMR, Lo–Mendell–Rubin likelihood ratio test. The bold values emphasize the selection of this model as the final fitted model.

Abbreviations: aBIC, adjusted Bayesian information criterion; AIC, Akaike information criterion; BIC, Bayesian information criterion; BLRT, bootstrapped likelihood ratio test.

**Table 3:** Profiles differences in professional quality of life.

Variables	Profile 1	Profile 2	*t*	*p*
Compassion satisfaction (CS)	34.47 ± 6.23	41.81 ± 6.52	−26.99	< 0.001
Burnout (BO)	24.86 ± 5.18	19.00 ± 5.73	25.04	< 0.001
Secondary traumatic stress (STS)	25.38 ± 5.94	22.54 ± 6.47	10.67	< 0.001

**Table 4:** Comparison of professional quality of life among nurses with different demographic characteristics (*N* = 2253).

Variables	CS	BO	STS
*Gender*			
Male	37.81 ± 7.90	22.83 ± 6.74	24.82 ± 7.62
Female	38.70 ± 7.31	21.44 ± 6.17	23.69 ± 6.30
*t*	−1.430	2.651	2.073
*p*	0.153	0.008	0.038

*Age (years)*			
18–30	36.51 ± 7.33	22.66 ± 6.21	23.56 ± 6.01
31–45	39.65 ± 7.16	21.03 ± 6.19	23.87 ± 6.71
46–60	40.56 ± 4.76	20.16 ± 5.59	23.92 ± 5.30
*F*	51.398	20.565	0.624
*p*	< 0.001	< 0.001	0.536

*Marital status*			
Unmarried	36.30 ± 7.27	22.84 ± 6.04	23.63 ± 6.08
Married	39.55 ± 7.18	21.05 ± 6.22	23.88 ± 6.55
Divorced/widowed	39.40 ± 7.35	20.32 ± 5.94	22.21 ± 5.56
*F*	45.739	20.087	2.077
*p*	< 0.001	< 0.001	0.126

*Number of children*			
0	36.48 ± 7.31	22.80 ± 6.08	23.74 ± 6.16
1	39.74 ± 7.04	20.83 ± 6.08	23.76 ± 6.47
2	40.43 ± 7.18	20.66 ± 6.43	23.86 ± 6.78
3	37.00 ± 5.89	22.14 ± 8.26	24.00 ± 4.55
*F*	41.366	18.861	0.034
*p*	< 0.001	< 0.001	0.992

*Education level*			
Junior college	37.52 ± 8.13	21.78 ± 3.88	23.14 ± 6.48
Undergraduate	38.84 ± 7.28	21.39 ± 6.17	23.77 ± 6.41
Graduate	37.28 ± 7.52	22.80 ± 6.16	24.14 ± 6.30
*F*	5.223	4.433	0.852
*p*	0.005	0.012	0.427

*Working years*			
1–10	37.33 ± 7.34	22.22 ± 6.16	23.72 ± 6.21
11–20	39.53 ± 7.31	21.18 ± 6.32	23.94 ± 6.75
21–30	40.67 ± 6.68	19.85 ± 5.62	23.05 ± 5.75
31–40	39.85 ± 6.73	20.83 ± 5.82	24.15 ± 5.74
F	21.488	10.189	1.123
*p*	< 0.001	< 0.001	0.339

*Working area*			
Internal medicine	38.32 ± 7.36	21.64 ± 6.21	23.95 ± 6.43
Surgery	38.92 ± 7.38	21.07 ± 6.21	23.08 ± 6.27
Intensive care unit	37.98 ± 7.35	22.41 ± 6.17	24.62 ± 6.39
Other wards	39.29 ± 7.28	21.17 ± 6.21	23.56 ± 6.43
*F*	3.333	4.437	4.868
*p*	0.019	0.004	0.002

*Rotational night shift*			
Yes	37.72 ± 7.56	22.24 ± 6.31	24.10 ± 6.49
No	40.20 ± 6.72	20.33 ± 5.87	23.22 ± 6.22
*t*	−7.851	7.134	3.174
*p*	< 0.001	< 0.001	0.002

*Note:* BO, burnout.

Abbreviations: CS, compassion satisfaction; STS, secondary traumatic stress.

**Table 5:** The effect of different latent profiles of team job crafting on professional quality of life in hierarchical linear regression.

**Dependent variable**	**Independent variable**	**Model 1**		**Model 2**	
		**Beta**	**p**	**Beta**	**p**
**CS**	**Constant**	**—**	**< 0.001**	**—**	**< 0.001**

	Age				
	18–30 (ref)				
	31–45	0.096	0.011	0.048	0.145
	46–60	0.060	0.099	0.042	0.195
	Marital status				
	Unmarried (ref)				
	Married	0.022	0.542	0.002	0.944
	Divorced/widowed	−0.002	0.948	−0.003	0.874
	Number of children				
	0 (ref)				
	1	0.128	0.001	0.093	0.009
	2	0.128	< 0.001	0.101	< 0.001
	3	−0.006	0.777	−0.014	0.445
	Education level				
	Junior college (ref)				
	Undergraduate	−0.019	0.559	−0.007	0.801
	Graduate	−0.059	0.067	−0.053	0.061
	Working years				
	1–10 (ref)				
	11–20	−0.034	0.288	−0.020	0.488
	21–30	−0.010	0.738	−0.008	0.758
	31–40	−0.035	0.264	−0.019	0.486
	Working area				
	Internal medicine (ref)				
	Surgery	0.050	0.033	0.031	0.136
	Intensive care unit	0.008	0.744	0.033	0.106
	Other wards	0.057	0.017	0.077	< 0.001
	Rotational night shift				
	Yes (ref)				
	No	0.090	< 0.001	0.057	0.008
	Team job crafting				
	Profile 1 (ref)				
	Profile 2			**0.466**	**< 0.001**
	*F*	10.686	< 0.001	638.997	< 0.001
	*R* ^2^	0.071		0.278	
	Adjusted *R*^2^	0.064		0.272	

**BO**	**Constant**	-	**< 0.001**	-	**< 0.001**

	Gender				
	Male (ref)				
	Female	−0.021	0.311		
	Age				
	18–30 (ref)				
	31–45	−0.050	0.192	−0.004	0.898
	46–60	−0.029	0.441	−0.011	0.736
	Marital status				
	Unmarried (ref)				
	Married	−0.001	0.979	0.019	0.571
	Divorced/widowed	−0.013	0.577	−0.012	0.586
	Number of children				
	0 (ref)				
	1	−0.109	0.008	−0.076	0.037
	2	−0.095	0.006	−0.070	0.025
	3	0.001	0.972	0.008	0.656
	Education level				
	Junior college (ref)				
	Undergraduate	0.031	0.343	0.079	0.505
	Graduate	0.085	0.010	−0.014	0.007
	Working years				
	1–10 (ref)				
	11–20	0.052	0.109	0.038	0.196
	21–30	0.004	0.880	0.002	0.926
	31–40	0.037	0.240	0.022	0.437
	Working area				
	Internal medicine (ref)				
	Surgery	−0.050	0.037	−0.031	0.142
	Intensive care unit	0.026	0.267	0.003	0.906
	Other wards	−0.026	0.292	−0.045	0.041
	Rotational night shift				
	Yes (ref)				
	No	−0.106	< 0.001	−0.073	< 0.001
	Team job crafting				
	Profile 1 (ref)				
	Profile 2			**−0.449**	**< 0.001**
	*F*	6.640	< 0.001	38.516	< 0.001
	*R* ^2^	0.046		0.237	
	Adjusted *R*^2^	0.039		0.231	

**STS**	**Constant**	**—**	**< 0.001**	-	**< 0.001**

	Gender				
	Male (ref)				
	Female	−0.032	0.136	−0.025	0.233
	Working area				
	Internal medicine (ref)				
	Surgery	−0.060	0.012	−0.053	0.023
	Intensive care unit	0.028	0.243	0.016	0.495
	Other wards	−0.021	0.379	−0.034	0.155
	Rotational night shift				
	Yes (ref)				
	No	−0.057	0.008	−0.031	0.144
	Team job crafting				
	Profile 1 (ref)				
	Profile 2			**−0.211**	**< 0.001**
	*F*	4.984	< 0.001	21.476	< 0.001
	*R* ^2^	0.011		0.054	
	Adjusted *R*^2^	0.009		0.052	

*Note:* BO, burnout. The bold values represent the influence of team job crafting's latent profiles on the quality of work life and enhance the clarity of the three dependent variables.

Abbreviations: CS, compassion satisfaction; STS, secondary traumatic stress.

## Data Availability

The data that support the findings of this study are available from the corresponding author upon reasonable request.
